# Analysis of Ocular Injury Characteristics in Survivors of the 8.12 Tianjin Port Explosion, China

**DOI:** 10.1155/2019/1360805

**Published:** 2019-08-07

**Authors:** Hao Jiang, Chao Xue, Yanlin Gao, Yan Wang

**Affiliations:** ^1^Clinical College of Ophthalmology, Tianjin Medical University, Tianjin, China; ^2^Tianjin Eye Hospital & Eye Institute, Tianjin Key Lab of Ophthalmology and Visual Science, Tianjin 300020, China

## Abstract

**Introduction:**

On the evening of August 12, 2015, a large chemical explosion occurred at Tianjin Port. We analyzed ocular injury characteristics in the survivors of this accident.

**Methods:**

Twenty injured eyes of 17 hospitalized patients were included. Initial best-corrected visual acuity (BCVA), injury type, injury cause, relative afferent pupillary defect (RAPD), zone of injury (ZOI), and ocular trauma score (OTS) were evaluated. Final BCVA and enucleation were the final outcome index. The relationship between risk factors and final outcomes was analyzed.

**Results:**

The patients comprised 14 males and 3 females (mean age, 35.24 ± 12.68 years). Eighteen eyes had open-globe and 2 had closed-globe injuries. Fifteen ocular injury types were reported. Initial visual acuity (VA) was 20/50 to 20/200, 20/200 to finger counting (FC), hand motion to light perception (HM-LP), and no light perception (NLP) in 2, 7, 7, and 4 eyes, respectively. RAPD was found in 5 eyes. Most eyes sustained severe injuries with OTSs of 1 (25%) and 2 (40%). Of the injured eyes, 50% had Zone III injuries. In 95% of the injured eyes, glass was the cause of injury. Three of 4 eyes with an initial VA of NLP had a final VA of NLP and an outcome of enucleation. In 5 eyes with RAPD, 3 had a final VA of NLP and a final outcome of enucleation. Eyes with lower OTSs generally had poorer outcomes. All eyes with a final VA of NLP and an enucleation outcome had Zone III injuries. All 3 eyes with an enucleation outcome had retinal injuries, whereas eyes with no retinal injury had a better final BCVA.

**Conclusions:**

Explosions can inflict severe ocular trauma, even indoors; 90% of injured eyes had open-globe injuries caused by glass fragments. Initial NLP, RAPD, low OTS, posterior extended wound, and retinal injury indicate a poor final outcome.

## 1. Introduction

On the evening of August 12, 2015, a large chemical explosion occurred at Tianjin Port, resulting in 165 deaths, 8 missing persons, 798 hospitalizations, and extensive property loss. As the most severe non-war-related chemical explosion since the establishment of the People's Republic of China, this accident received extensive local and international attention.

Several studies have been conducted on this explosion accident. Zhang et al. [1] presented the responses of emergency medical services and hospitals to the explosion and summarized the lessons that can be learned. Li et al. [2] also analyzed the emergency medical response to this event and reported the experiences of trauma physicians to optimize the use of medical resources and reduce the mortality rate for critical patients. Guo et al. [3] analyzed the treatment process for the wounded and reported the experiences of the organization and management of emergency rescue operations. In addition, the study [3] revealed that ocular trauma was the most common injury resulting from the accident. However, the focus of these studies was on the organization and management of emergency medical resources. Despite being a major injury type, the ocular trauma caused by this explosion has not yet been reported.

As the largest eye hospital in Tianjin, we received the majority of eye injury victims after this accident. The purpose of this study was to present and discuss the ocular injuries caused by this explosion accident. We focused on the characteristics of the ocular injuries and the most severe risk factors that might affect the final outcomes.

## 2. Methods

This was a retrospective study including 20 injured eyes in 17 hospitalized patients treated in our hospital. Patients with relatively minor corneal or conjunctive foreign bodies, adnexal lacerations sutured in the outpatient department, or small hyphemas were not included. The medical injury data were collected from hospital records and physician surveys administered at the initial examination and additional surgeries.

The following items were evaluated in this study: type of injury, cause of injury, wound characteristics, relative afferent pupillary defect (RAPD), visual acuity, and associated ocular and ocular adnexal injuries. The zone of injury (ZOI) and ocular trauma score (OTS) were determined at the initial examination, and the data were recorded during the primary surgery. The ZOI was classified according to the Ocular Trauma Classification Group: Zone I (wound involvement limited to the cornea, including the corneoscleral limbus), Zone II (wound involving the sclera and no more posterior than 5 mm from the corneoscleral limbus), and Zone III (wound involvement posterior to the anterior 5 mm of the sclera) [4, 5]. The method of calculating OTS is summarized in Table 1 [6]. For eyes with hyphemas in which RAPD could not be observed clearly, the indirect pupillary light reflex of the fellow eye was examined instead.

Five risk factors (initial best-corrected visual acuity [BCVA], OTS, ZOI, RAPD, and retinal injury) and two outcomes (final BCVA and enucleation) were identified for analysis. Terms used in the description of ocular injuries conformed to the recommendations of the American Academy of Ophthalmology, the United States Eye Injury Registry, and the International Society of Ocular Trauma [5].

This study was approved by the Ethics Committee of the Tianjin Medical University Eye Hospital. Informed consent was obtained from the patients and their families before any examination or treatment was performed.

## 3. Results

### 3.1. Characteristics of the Injuries

Twenty eyes in 17 patients were included in this study, with 3 patients having bilateral eye injuries. The patients comprised 14 men and 3 women, with a mean age of 35.24 ± 12.68 years (range, 23 to 64 years). All 17 patients were indoors at the moment of the explosion. The causes of the injuries were identified as being uncertain in one case, doorframe and glass fragments in another case, and glass fragments in the other 18 cases.

**Figure 1:**
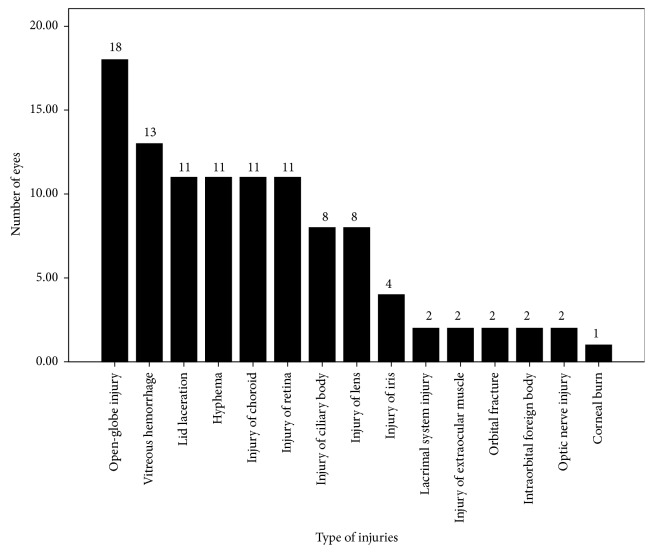
Types of ocular and adnexal injuries.

Two eyes had closed-globe injuries (corneal lamellar laceration) and 18 had open-globe injuries. Fifteen types of ocular injuries were reported. The most common injuries were open-globe injuries, vitreous hemorrhage, lid or brow lacerations, hyphemas, and choroidal and retinal injuries. The types of ocular and ocular adnexal injuries are listed in Figure 1. Some eyes had multiple injury types. An open-globe injury was defined as a full-thickness laceration of the cornea, sclera, or both [7]. Injury of the lens was defined as traumatic cataracts or dislocation or absence of the lens. Choroidal injury included suprachoroidal hemorrhage, choroidal rupture, choroidal detachment, and extrusion of the choroid. Retinal injury involved detachment of or holes in the retina.

Table 2 shows the characteristics and final outcomes of the 20 injured eyes. Initial visual acuity was 20/50 to 20/200, 20/200 to finger counting (FC), hand motion to light perception (HM-LP), and no light perception (NLP) in 2 (10%), 7 (35%), 7 (35%), and 4 (20%) eyes, respectively. RAPD was negative in 12 eyes. Indirect pupillary light reflex of the fellow eye was not found in 5 of the other 8 eyes, which had hyphemas.

The majority of the eyes sustained severe injuries with OTSs of 1 (25%) and 2 (40%). Of the remaining patients, 20% had an OTS of 3 and 15% had an OTS of 4. None of the patients had an OTS of 5. In terms of the ZOI, 10 of the injured eyes had Zone III injuries, 6 (30%) had Zone I injuries, and 4 (20%) had Zone II injuries.

### 3.2. Special Examinations and Treatment

**Figure 2:**
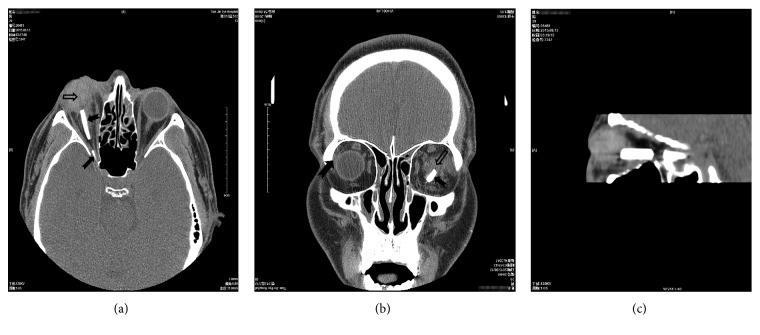
Computed tomography scan of a patient exhibiting eyeball rupture with vitreous hemorrhage (indicated by hollow arrows) and foreign body at the orbital and superior orbital fissure (indicated by solid arrows). (a) Axial scanning. (b) Coronal scanning. (c) Sagittal scanning.

**Figure 3:**
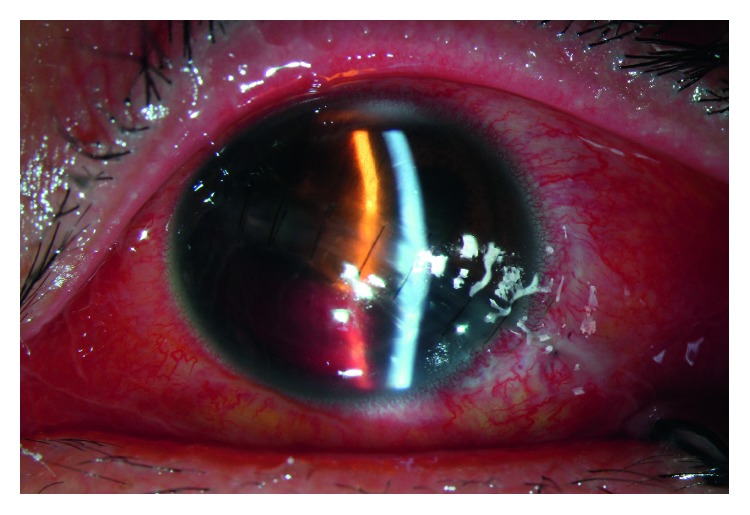
Slit-lamp photograph of a patient 1 week after repair of the globe.

**Figure 4:**
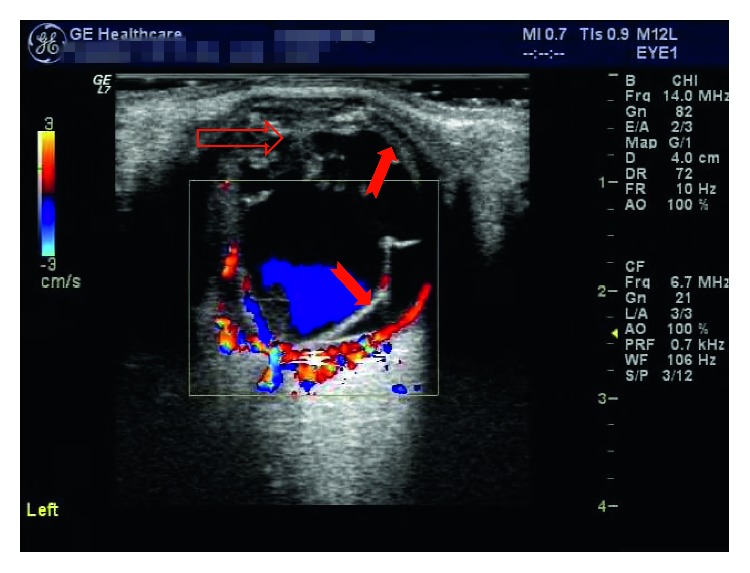
Color Doppler ultrasonography of a patient exhibiting vitreous fibroplasias (indicated by hollow arrows) and choroidal and retinal detachment (indicated by solid arrows) 3 days after repair of the globe.

In addition to routine examination, computed tomography (CT) scans were performed in each patient before surgery to confirm eyeball rupture, intraorbital and/or intraocular foreign bodies, or orbital and/or optic canal fracture. Electroretinography, multifocal electroretinography, and visual evoked potential examinations were employed to evaluate the condition of the retina and optic nerve, as required. B-scan ultrasonography or color Doppler ultrasonography was performed to assess the condition of the vitreous body, choroid, and retina at appropriate times. Ultrasound biomicroscopy was used to determine the anterior segment condition of the eye, as required. Figures 2–4 show examination results.

Sixteen of the 20 eyes underwent surgery within 24 h after injury to repair the globe, and 3 eyes that did not undergo surgery were treated with therapeutic contact lenses. In the eyes that underwent surgery, 2 eyes underwent 4 surgeries, 3 eyes underwent 3 surgeries, 5 eyes underwent 2 surgeries, and 6 eyes underwent 1 surgery. In the patients who underwent 2 surgeries, the second surgery comprised vitrectomy and silicone oil injection. The third surgery was enucleation for 3 eyes and silicone oil injection for 2 eyes. The fourth surgery was a simple silicone oil injection and epiretinal membrane peeling with a secondary silicone oil injection for 2 eyes. In addition, 2 eyes received retinal laser photocoagulation after repair of the globe.

### 3.3. Final Outcomes

Final visual acuity was ≤20/40, 20/50 to 20/200, 19/200 to FC, HM to LP, and NLP in 8 (40%), 5 (25%), 3 (15%), 1 (5%), and 3 (15%) eyes, respectively. Three of the 20 eyes had a final outcome of enucleation.

### 3.4. Relationship between Risk Factors and Final Outcomes

In the 4 eyes with a primary visual acuity of NLP, only 1 injured eye had a final outcome of light perception (LP); the final visual acuity of the other 3 eyes was NLP with an outcome of enucleation. In 5 eyes with a positive RAPD, 4 of which had an initial visual acuity of NLP and the other had an initial visual acuity of LP, whereas 3 had a final visual acuity of NLP and the other 2 had final visual acuities of LP and HM. Three of these 5 eyes had a final outcome of enucleation.

All of the eyes with a final visual acuity of NLP and LP had an OTS of 1. In addition, all 3 eyes with a final outcome of enucleation also had an OTS of 1. Eight eyes with an OTS of 2 had a final visual acuity, ranging from FC to 20/20. Four eyes with an OTS of 3 had a better final visual acuity, ranging from 20/100 to 20/20. The other 3 eyes with an OTS of 4 had final visual acuities of 20/30, 20/20, and 20/20. In general, the eyes with a lower OTS had a poorer outcome. All of the eyes with a final visual acuity of NLP and an enucleation outcome had Zone III injuries.

Nine eyes with no retinal injury had a favorable final BCVA, ranging from 20/100 to 20/20. In another case (no. 19) with a minor retinal hole, the final BCVA was 20/20. In the other 10 eyes, with retinal injuries, the final BCVA ranged from NLP to 20/50. All 3 eyes with an enucleation outcome had retinal injuries.

Another remarkable result was that all 3 eyes with an enucleation outcome had an initial visual acuity of NLP, a positive RAPD syndrome, and Zone III injuries. In addition, all 3 eyes with retinal injuries had an OTS of 1.

## 4. Discussion

The incidence of ocular trauma from blast injuries sustained during disasters and combat has steadily increased in recent decades. The Oklahoma bombing in 1995 [8], World Trade Center terrorist attack in 2001 [9, 10], Madrid train bombings in 2004 [11], and London subway bombings in 2005 [12] were all mass-casualty incidents. Despite the ocular surface comprising only 0.10% of the total body surface area and 0.27% of the anterior silhouette, as much as 10% of blast survivors sustain ocular injuries [8].

Mines et al. [8] found that 8% of the injured bombing survivors sustained an ocular injury. In another study by Mader et al. [7], they found that 10% of all surgical admissions suffered severe ocular or ocular adnexal injuries. However, in this study, 17 of 798 hospitalizations had severe ocular or ocular adnexal injuries; the rate was 2.13% and was small compared with other studies. The reason may be that the explosion happened at midnight when most people were indoors in sleep. Another reason was that patients with relatively minor ocular injuries were not included in this study. In the study of Mader et al. [7], there were 44 bilateral ocular injuries for a total of 251 severe injuries; the rate of bilateral involvement was 17.53%. In our study, 17.64% had bilateral involvement, which was close to that in the Mader et al.

The mechanism of blast injuries has traditionally been divided into 4 categories: primary, secondary, tertiary, and quaternary. A primary blast injury is the direct result of the blast wave itself; air-filled structures, such as the lungs, eardrums, and gastrointestinal system, are most susceptible to primary blast injuries. Secondary blast injuries are caused by flying debris or fragments propelled by the explosion. Often exposed and unprotected, the eyes are particularly susceptible to secondary blast injuries. A tertiary injury is caused by displacement of the whole body or part of the body by the blast or structural collapse and fragmentation of a building. Quaternary blast injuries refer to other explosion-related injuries, including chemical or thermal burns, toxic inhalation, radiation exposure, and asphyxiation [8, 13, 14].

We spared no effort in repairing the structure and recovering the visual potential of every injured eye, even if it seemed to have minimal visual potential. Only functionally destroyed eyes with no possibility of visual or cosmetic salvage were removed. However, 35% of the injured eyes had a final BCVA lower than 20/200, and 15% of the injured eyes had a final outcome of enucleation. This eye excision rate was close to that reported in previous studies [7, 15]. Morley et al. [14] indicated that both severe penetrating and nonpenetrating blast-related eye injuries may be associated with a poor visual prognosis because of severe anterior and posterior segment damage.

In this study, the cause of almost all of the injuries was a single glass fragment, except in one case in which the cause was uncertain and another case in which the cause was doorframe and glass fragments. Epidemiological studies on explosions in modern buildings frequently cite glass as a major source of lacerations and foreign bodies affecting the eye [13]. Mines et al. [8] found that most injuries among the survivors of bombings result from secondary effects of the blast, in the form of flying and falling glass, building materials, and other debris. In a study conducted by Thach et al. [16], glass fragments caused by a blast were the mechanism of all the ocular injuries in the survivors. Our results are consistent with those of these previous studies.

In early hospital treatment, obtaining an objective measure of best visual acuity is critical as a “vital sign” of the eye. Initial BCVA appears to be the single most crucial factor in predicting final visual acuity in patients with penetrating injuries, with NLP being an indicator of poor prognosis [14]. Page et al. [17] found that preoperative BCVA was positively associated with final BCVA and enucleation. In the present study, 3 of 4 injured eyes (75%) with a primary visual acuity of NLP had a final outcome of NLP with an outcome of enucleation, and only 1 injured eye (15%) had a final outcome of LP. This finding constitutes further evidence that initial visual acuity of NLP is an indicator of poor prognosis.

An irregular pupillary response may indicate intracranial pathology [13]. The evaluation of RAPD in the injured eye is a crucial functional test and parameter for quantifying the loss of neuronal function in asymmetric optic nerve disease and correlates closely with estimated retinal ganglion cell loss in optic nerve disease [18]. As previous studies have reported, the presence of RAPD is critical in terms of predicting outcome and has been shown to be an independent prognostic factor of poor final visual acuity [19–22]. In this study, the presence of RAPD was found to be associated with a poor outcome. Three of 5 eyes (60%) with RAPD had a final visual acuity of NLP and a final outcome of enucleation. The other 40% (2 of 5 eyes) without RAPD had a final visual acuity of LP and HM. However, the tendency to neglect the importance of RAPD during the initial examination of injured eyes is concerning. Performing RAPD examination may be time-consuming but is critical as a routine practice.

The OTS was developed by Scott to predict visual outcome on the basis of an initial examination [6]. OTSs range from 1, indicating the most severely injured eyes, to 5, indicating the least injured eyes. The OTS has been widely used for the evaluation of eye injuries and has been proven to be a reliable indicator for predicting the visual outcome of various ocular traumas [23–25]. In general, eyes with a lower OTS had a poorer outcome in this study, which is in agreement with previous studies [17, 21, 26].

The ZOI is another factor likely to affect the outcome of open-globe injuries. In this study, all of the eyes with a final visual acuity of NLP with an enucleation outcome had Zone III injuries. This result is consistent with those from previous studies [22, 27] in which the results revealed that the more posteriorly the wound extends, the greater the probability of poor visual outcome is.

The relationship between the final outcome and injury combined with choroidal and retinal injury was investigated. Retinal detachment was found to be a crucial prognostic factor in previous studies [21, 28, 29]. When retinal detachment occurs, photoreceptor cells are likely to be severely injured, which may lead to a poor final visual acuity [27]. In the present study, all of the eyes with no retinal injury had a more favorable final BCVA, ranging from 20/100 to 20/20, whereas in the eyes with retinal injuries, 90.0% (10 of 11) had a final BCVA, ranging from NLP to 20/50. The only exception was an injured eye with a minor retinal hole and a final BCVA of 20/20. In addition, all 3 eyes with an enucleation outcome had retinal injuries.

The time between the injury and primary surgery has also been reported to be crucial in determining prognosis, and patients who experience more prolonged treatment delays have been determined to have a higher rate of complications and subsequent enucleations [14, 17, 30]. Because all of the injured eyes underwent surgery within 24 h to repair the globe and the data were limited to a narrow range, we did not include treatment delay as a prognostic factor in this study.

This study had several limitations. As an eye hospital, we did not receive any ocular trauma patients who had severe injuries of other parts of the body and/or systemic diseases; therefore, the study may have failed to include patients admitted to other general hospitals. Another limitation was that statistical analysis was not performed because of the small sample size. Despite this limitation, the results showed the relationship between numerous risk factors and the final outcomes of eye trauma.

In summary, as the foremost ophthalmology hospital in the Tianjin region, we received the majority of survivors of the 8.12 Tianjin Port explosion accident who sustained ocular injuries. Of the injured eyes, 90% had open-globe injuries caused by glass fragments. Although we tried our best to treat and save the injured eyes, 15% had a final BCVA of NLP with an enucleation outcome. An initial visual acuity of NLP, presence of RAPD, a low OTS, a posteriorly extending wound, and retinal injury might indicate a poor final outcome.

## Figures and Tables

**Table 1:** Method for deriving the OTS.

*Variables*		
A: initial raw score (based on initial visual acuity)	Raw points	
	NLP =	60
	LP/HM =	70
	1/200–19/200 =	80
	20/200–20/50 =	90
	≥20/40 =	100
B: rupture		−23
C: endophthalmitis		−17
D: perforating injury		−14
E: retinal detachment		−11
F: RAPD		−10
*Conversion of raw points into OTS category*		
Raw score sum		OTS score
0–44		1
45–65		2
66–80		3
81–91		4
92–100		5

OTS: ocular trauma score; NLP: no light perception; LP: light perception; HM: hand motion; RAPD: relative afferent pupillary defect.

**Table 2:** Characteristics and final outcomes of 20 injured eyes.

No.	Initial BCVA	RAPD	Retinal injury	OTS	ZOI	Surgeries/other therapies performed	Final BCVA	Enucleation
1	FC	−	−	3	II	1^st^: ocular repairing; 2^nd^: vitrectomy + SO injection	20/30
2	20/200	−	−	4	I	Wearing therapeutic contact lenses	20/20	
3	NLP	+	+	1	III	1^st^: ocular repairing; 2^nd^: vitrectomy + SO injection; 3^rd^: enucleation	NLP	+
4	LP	−	+	2	III	1^st^: ocular repairing; 2^nd^: vitrectomy + SO injection; 3^rd^: enucleation	20/50	
5	NLP	+	+	1	III	1^st^: ocular repairing; 2^nd^: retinal laser photocoagulation	NLP	+
6	NLP	+	+	1	I	1^st^: ocular repairing; 2^nd^: vitrectomy + SO injection	LP	
7	HM	−	−	2	II	Ocular repairing	20/30	
8	20/200	−	−	3	II	Ocular repairing	20/100	
9	20/50	−	−	4	I	Eye drops therapy	20/30	
10	20/200	−	−	3	I	Wearing therapeutic contact lenses	20/20	
11	20/50	−	−	4	I	Wearing therapeutic contact lenses	20/20	
12	FC	−	+	2	III	1^st^: ocular repairing; 2^nd^: vitrectomy + SO injection	FC	
13	LP	−	+	2	III	1^st^: ocular repairing; 2^nd^: vitrectomy + SO injection; 3^rd^: SO injection; 4^th^: SO injection	FC	
14	LP	−	+	2	III	1^st^: ocular repairing; 2^nd^: vitrectomy + SO injection; 3^rd^: SO injection; 4^th^: SO injection	20/60	
15	20/300	−	−	3	I	Ocular repairing	20/60	
16	LP	−	−	2	II	Ocular repairing	20/40	
17	NLP	+	+	1	III	1^st^: ocular repairing; 2^nd^: vitrectomy + SO injection; 3^rd^: enucleation	NLP	+
18	LP	−	+	2	III	1^st^: ocular repairing; 2^nd^: vitrectomy + SO injection	20/200	
19	20/200	−	+	2	III	1^st^: ocular repairing; 2^nd^: retinal laser photocoagulation	20/20	
20	LP	+	+	1	III	1^st^: ocular repairing; 2^nd^: vitrectomy + SO injection	FC	

BCVA: best-corrected visual acuity; RAPD: relative afferent pupillary defect; OTS: ocular trauma score; ZOI: zone of injury; FC: finger counting; NLP: no light perception; LP: light perception; HM: hand motion; SO: silicone oil.

## Data Availability

The data used to support the findings of this study are available from the corresponding author upon request.
